# Mechanisms of pathogenicity for the emerging fungus *Candida auris*

**DOI:** 10.1371/journal.ppat.1011843

**Published:** 2023-12-21

**Authors:** Mark V. Horton, Ashley M. Holt, Jeniel E. Nett

**Affiliations:** 1 Department of Medicine, University of Wisconsin, Madison, Wisconsin, United States of America; 2 Department of Medical Microbiology & Immunology, University of Wisconsin, Madison, Wisconsin, United States of America; Universitat Zurich, SWITZERLAND

## Abstract

*Candida auris* recently emerged as an urgent public health threat, causing outbreaks of invasive infections in healthcare settings throughout the world. This fungal pathogen persists on the skin of patients and on abiotic surfaces despite antiseptic and decolonization attempts. The heightened capacity for skin colonization and environmental persistence promotes rapid nosocomial spread. Following skin colonization, *C*. *auris* can gain entrance to the bloodstream and deeper tissues, often through a wound or an inserted medical device, such as a catheter. *C*. *auris* possesses a variety of virulence traits, including the capacity for biofilm formation, production of adhesins and proteases, and evasion of innate immune responses. In this review, we highlight the interactions of *C*. *auris* with the host, emphasizing the intersection of laboratory studies and clinical observations.

## Scope of the public health threat

Since the initial description of this new species in 2009, *Candida auris* has caused devasting outbreaks of difficult-to-treat infections in healthcare facilities spanning 6 continents [1–8]. *C*. *auris* appears to have emerged relatively independently in separate locations and isolates cluster into at least 4 distinct geographic clades [2,9,10]. *C*. *auris* effectively colonizes the skin of patients, particularly those with extended hospital stays and prior antibiotic or antifungal exposure [11–13]. A subset of these patients can develop invasive infection, often in the setting of indwelling medical devices, feeding tubes, and other surgical procedures [11–13]. *C*. *auris* isolates exhibit high rates of drug resistance to common classes of antifungals, often narrowing treatment options [14,15]. Mortality rates vary across studies, but reports as high as 60% have been documented [2]. Due to these factors, the Centers for Disease Control and Prevention classifies drug-resistant *C*. *auris* as an urgent threat, placing the organism in the most serious category [16].

Although *C*. *auris* infections tend to cluster as epidemic outbreaks, the overall cases have continued to rise in recent years. Reports can include cases of infection, where *C*. *auris* is isolated from a site of infection, or cases of colonization, where *C*. *auris* is identified on the skin or other site and not producing symptomatic infection. In the United States, cases of *C*. *auris* skin colonization nearly tripled from 1,310 cases in 2020 to 4,041 cases in 2021 [17]. Cases of invasive candidiasis and candidemia due to *C*. *auris* are also on the rise in many areas. For example, a report examining critically ill patients in India with candidemia found *C*. *auris* to be the most common species, responsible for approximately 40% of all cases [18]. A study from Kuwait also reported an increasing proportion of *C*. *auris* bloodstream isolates (13.7%) compared to years prior [19]. In the US, the number of invasive clinical infections of *C*. *auris* also rose sharply, nearly doubling from 756 cases in 2020 to 1,471 cases in 2021 [17]. Clinical investigations have additionally questioned the likelihood of developing an invasive infection after *C*. *auris* establishes skin colonization. For patients previously known to harbor *C*. *auris* on skin, the rate of progression to candidemia ranges from <25% to 74.5% [20,21]. These findings point to the need for greater understanding of skin colonization not only to combat *C*. *auris* growth on skin but also to prevent subsequent invasive infections.

## Skin colonization by *C*. *auris*

Multiple clinical studies have analyzed the prevalence of *C*. *auris* skin colonization in outbreak settings and have reported colonization for 37.5% to 86% of participants [4,22–25]. *C*. *auris* skin colonization cases appear to be increasing in recent years, paralleling the rise of invasive disease. For example, reported rates of skin colonization in the US increased over 200% in 2021 compared to 2020 [17]. Common sites of skin colonization sampling include the axilla and groin, but a recent work pointed to the importance of expanding the sampling areas to other highly colonized areas including nares, fingertips/palms, toe web, and perianal area [25]. Of note, oral *C*. *auris* colonization has not been commonly noted for patients. This is consistent with reports of poor oral colonization in mice and *C*. *auris* susceptibility to the salivary antimicrobial peptide histatin 5 [26,27]. Single-site testing found that nares was the most sensitive testing site for determining colonized patients (53.1% sensitivity), and the combination of nares with palm and fingertips yielded the highest 2-site sensitivity (76.1% sensitivity) [25]. The colonization of the palms/fingertips of patients is particularly concerning for efficient spread person-to-person or via high touch surfaces [25]. *C*. *auris* may also spread via contaminated gloves. In laboratory studies, viable colonies of *C*. *auris* could be recovered from fingertips of both latex and nitrile gloves, as well as from a urinary catheter surface after encounter with wet or dried contaminated gloves [28]. It is likely that the ability of *C*. *auris* to remain viable under dry conditions on numerous abiotic surfaces contributes to potential contamination of catheters, other medical devices, and shared medical equipment [23,29–31]. Hand hygiene remains a critical component of controlling outbreaks.

As high-burden skin colonization poses significant risk for hospital transmission and development of invasive infection, there has been great interest in exploring methods to decolonize skin. Bathing patients with a 2% solution of chlorhexidine gluconate (CHG) is a common approach for the cleansing of patient skin in many clinical settings, including intensive care units. In vitro studies show that *C*. *auris* isolates are inhibited by CHG at <0.02% [32,33]. However, despite this in vitro activity, *C*. *auris* can persist on patient skin in healthcare facilities that implement routine CHG bathing [23,25,34]. The reasons for this appear multifactorial. Routine bathing may not adequately distribute CHG to all colonized sites. In studying CHG bathing for colonized patients in a skilled nursing facility in Illinois, Proctor and colleagues found that fewer than 10% of skin sites received concentrations sufficient to reduce the odds of *C*. *auris* colonization, with the minimal concentration associated with significantly reduced colonization calculated to be 625 μg/ml [25]. This calculated CHG concentration is equivalent to approximately 0.6%, 20 to 39 times higher than the CHG concentration noted for growth inhibition of *C*. *auris* in vitro, suggesting decreased effectiveness on skin compared to in vitro conditions [25,32,33].

Animal models of *C*. *auris* skin colonization recapitulate the limitations of CHG treatment for decolonization of skin. In a murine skin model for prevention of *C*. *auris* colonization, preexposure and ongoing treatment with 2% CHG wipes could prevent colonization following low inoculum (10^7^ CFU) exposure. However, treatment reduced but did not fully prevent *C*. *auris* colonization upon exposure to higher burden (10^9^ CFU) [35]. When CHG was used as a treatment for established *C*. *auris* colonization, the viable burden on the skin surface decreased but was not fully eradicated. Furthermore, CHG treatment minimally impacted the viable burdens of deeper skin samples [35]. Similar observations were found using an ex vivo porcine skin model where 2% CHG treatment of *C*. *auris* on skin resulted in a meager 0.5 log-reduction in viable yeast [36]. While CHG treatment reduces *C*. *auris* growth on skin, it does not appear to eliminate it completely. *C*. *auris* likely persists in deeper tissues and follicles, allowing it the opportunity to proliferate [35,36].

**Fig 1:**
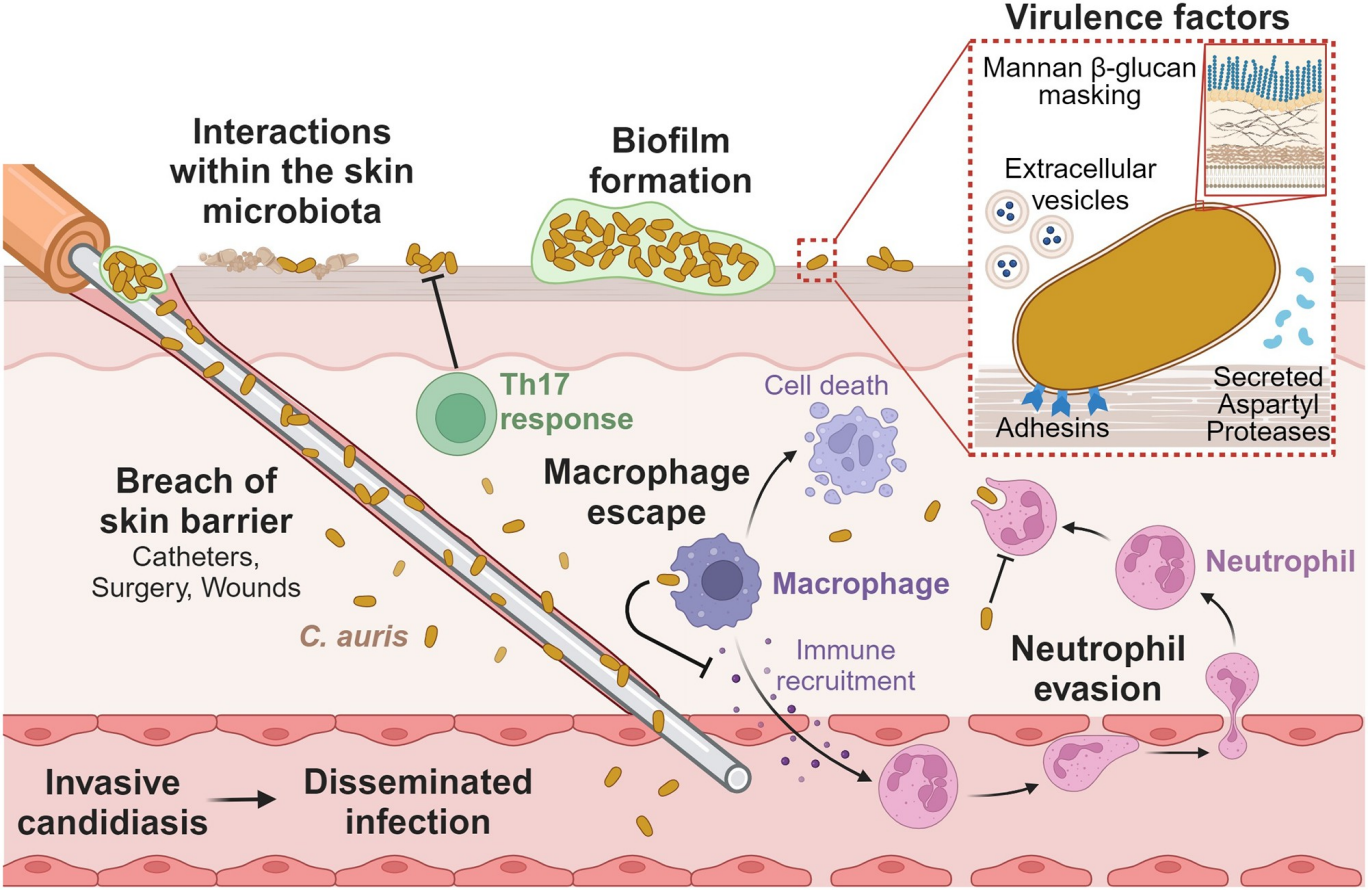
*Candida auris* mechanisms of pathogenesis. *C*. *auris* colonizes and persists on the skin, and breaches of the skin barrier can lead to invasive candidiasis and hematogenous spread of infection. Interactions with other skin microbiota (e.g., *Malassezia* spp., *Klebsiella pneumoniae*, *Staphylococcus hominis*) influence the growth of *C*. *auris* on the skin. Biofilm formation of *C*. *auris* increases resistance to antimicrobial treatment on the skin surface. *C*. *auris* produces multiple virulence factors to release into the surrounding microenvironment including SAPs and EVs. Adhesins promote attachment to the skin surface, and mannans in the cell wall mask β-glucans from PRRs on immune cells. *C*. *auris* evades neutrophil recognition and phagocytosis. *C*. *auris* escapes macrophage killing, inhibits macrophage-mediated immune recruitment, and induces macrophage cell death. In contrast, the Th17 T-cell response inhibits *C*. *auris* skin colonization. The figure was designed using Biorender. EV, extracellular vesicle; PRR, pathogen recognition receptor; SAP, secreted aspartyl protease.

*C*. *auris* exhibits the capacity to form biofilms during skin colonization (Fig 1) [37]. Growth in this form may further limit the activity of CHG, as in vitro biofilms (when compared to planktonic cells) are approximately 10-fold more resistant to the activity of CHG, and are not fully eradicated by treatment with 2% CHG [36,38]. Ex vivo studies suggest that the addition of 70% isopropyl alcohol and commonly used topical essential oils, including tea tree (*Melaleuca alternifolia*) oil and lemongrass (*Cymbopogon flexuosus*) oil, can improve the activity of CHG [36]. Study using a guinea pig model of skin colonization also suggests that the addition of systemic antifungal therapy may also help to decrease burden of skin colonization [39]. Given the lack of clinical data to show successful decolonization, there are currently no CDC-recommended strategies to eradicate *C*. *auris* from skin (https://www.cdc.gov/fungal/candida-auris/c-auris-infection-control.html).

Acquisition of *C*. *auris* correlates with prior receipt of antibiotics and antifungals, suggesting a role for skin dysbiosis [11–13,25]. Proctor and colleagues examined the skin microbiome of patients with and without *C*. *auris* colonization. They found that patients with skin mycobiomes dominated by *Malassezia* species were at a lower risk of *C*. *auris* dominance on the skin [25]. Other mycobiomes were primarily dominated by a diverse group of *Candida* spp., which appeared to represent a transitional state of the skin mycobiome. Mycobiomes with shared dominance by a variety of *Candida* species switched to a singular dominance by *C*. *auris* 30% to 50% of the time. These data highlight the importance of studying *Malassezia* species during interactions within the skin microbiome (reviewed in [40]). The group also examined bacterial communities and identified organisms with higher abundance in *C*. *auris*-colonized patients (*Proteus mirabilis*, *Klebsiella pneumoniae*, *Providencia stuartii*, and *Pseudomonas aeruginosa*). In contrast, *Staphylococcus hominis* was more abundant in the patients without *C*. *auris* colonization. Further understanding of how the skin microbiome influences *C*. *auris* may help to identify strategies to prevent or eliminate colonization. For example, cleansing methods that promote healthy skin microbiota may help to decrease the rate of *C*. *auris* skin colonization.

## Adhesion, biofilm formation, and environmental persistence

*C*. *auris* survives in harsh conditions on both nonliving and living surfaces in healthcare environments. *C*. *auris* has been isolated from multiple sources in hospitals including floors, bed rails, bed sheets, door handles, oxygen masks, and sinks [4,22]. The adaptability of *C*. *auris* for growth on a variety of abiotic surfaces has been described in laboratory studies, demonstrating survival of *C*. *auris* on plastic for multiple weeks in low-moisture environments [29,30,41]. A unique profile in withstanding salt stress and osmotic stress may play an addition role in long-term environmental persistence on surfaces in the healthcare settings [42,43]. Biofilm formation likely contributes to *C*. *auris* environmental persistence and tolerance of biocides. Clinical isolates form in vitro biofilms in tissue culture media (RPMI) on plastic with some heterogeneity in density noted among clades and isolates [27,44–46]. Even more dense biofilms form in synthetic skin/sweat media, suggesting that devices in contact with skin may be particularly prone to contamination by *C*. *auris* biofilms [37,47]. *C*. *auris* biofilms formed in this milieu can survive desiccation for weeks [37]. Kean and colleagues further showed that the biofilm phenotype promotes resistance to antiseptics, including H_2_O_2_, povidone iodine, and CHG, additionally contributing to persistence in healthcare settings [38]. Biofilms formed during skin colonization are expected to be highly tolerant to these therapies, as is observed clinically for CHG [23,25,34].

Surface adhesion is a critical first step for biofilm formation and skin colonization. For *C*. *albicans* and other *Candida* spp., roles for specific adhesins have been well described [48–50]. Members of the agglutin-like sequence (ALS) family of adhesins function in adherence and as virulence factors in *Candida* species and homologs to *ALS* family proteins have been identified in *C*. *auris* [51,52]. The roles of these adhesins appear to vary among isolates and across the *C*. *auris* life cycle. Using a 3D in vitro wound infection model, Brown and colleagues examined transcriptional patterns associated with biofilm formation [53]. They found *ALS5* to be associated with biofilm formation for isolates with an aggregative/clustering growth phenotype, but not for those lacking this phenotype, suggesting a role for *ALS5* during biofilm growth for these isolates. This study and others have noted heterogeneity in biofilm formation across clinical isolates [45,54], with one potential factor being the lack of cell wall and *ALS*-like genes in Clade II strains [55]. This finding may aid in explaining heterogeneity in biofilm formation, lack of outbreaks for Clade II isolates, and lower skin colonization of Clade II strains in a mouse model [35,55]. Bing and colleagues found that expression of *ALS4* across *C*. *auris* isolates correlated with protein-dependent aggregation and heightened biofilm formation [56,57]. Whole genome analysis revealed genomic amplification for *ALS4* with high copy number variations among clinical isolates. The replicative age of *C*. *auris* also influences expression of adhesins, where older *C*. *auris* cells show increased expression of *ALS5* and exhibit thicker cell walls [58].

*C*. *auris* has also been shown to express at least one unique adhesin. Santana and colleagues identified the previously uncharacterized adhesin Scf1 with homologs only in *C*. *auris* and closely related strains of *C*. *haemulonii* [59]. Among *C*. *auris* isolates included in the study, transcriptional abundance of *SCF1* positively correlated with adhesion of the isolates. Loss of *SCF1 and IFF4109*, a conserved member of the IPF Family F/Hyphally Regulated adhesin family, in *C*. *auris* strain resulted in decreased fungal loads in an immunocompromised mouse model of disseminated infection and decreased ability to colonize ex vivo human skin and proliferate on the luminal surface of a polyethylene rat central venous catheter. Scf1 interactions were linked to exposed cationic residues, in contrast to the hydrophobic interactions typically observed for fungal adhesions. This novel adhesin appears to contribute to the capacity of *C*. *auris* to colonize skin, form biofilm-associated infections, and persist on surfaces.

It is interesting to speculate how the skin microbiota may influence *C*. *auris* adhesion, biofilm formation, and skin colonization. Patients with skin mycobiomes dominated by *Malassezia* species appear to be at a lower risk of *C*. *auris* dominance on the skin [25]. Similarly, *Staphylococcus hominis* abundance negatively correlates with *C*. *auris* colonization. Mechanistic insight into these interactions is limited. However, fungi, including common probiotic strains *Saccharomyces cerevisiae* and *Issatchenkia occidentalis*, have been shown to reduce *C*. *auris* adhesion to plastic by up to 60% [60]. It is possible that skin microbiota modulate *C*. *auris* adherence properties.

## Catheters, breach of skin, and other risk factors for *C*. *auris*-invasive disease

Patients with *C*. *auris* skin colonization become increasingly at risk for invasive disease when there is a breach of the skin barrier that can serve as a portal of entry to the bloodstream and deeper tissues. For example, medical device implantation, recent surgery, total parenteral nutrition (TPN) administration, and catheter placement are risk factors for development of invasive *C*. *auris* infection in adult populations [11,13,61]. Similarly, a study of pediatric patients with *C*. *auris* bloodstream infection found that 82% had undergone central venous catheter placement, and 56% had received TPN [5]. To better understand and potentially predict which skin-colonized patients may progress to develop invasive disease, Garcia-Bustos and colleagues used clinical and epidemiological factors from a single hospital outbreak to construct a scoring system [11]. This study provided a model to estimate the factors posing the greatest risk for *C*. *auris* candidemia. They found that TPN to be the greatest risk factor, with recent surgery, central venous catheter placement, and arterial catheter placement among the other independent predictors of candidemia. Catheters likely serve as both a substrate for biofilm formation and a bridge from the epidermal surface to bloodstream (Fig 1). Furthermore, the nutritional components of TPN may promote fungal growth. When compared to other *Candida* spp., *C*. *auris* may have a heightened capacity to cause catheter-associated candidemia, perhaps due to its propensity for skin colonization. Retrospective analysis examining patients with candidemia found that those with *C*. *auris* candidemia were more likely to carry the diagnosis of catheter-associated bloodstream infection (89%) than those with candidemia due to other species (47%) [61].

**Fig 2:**
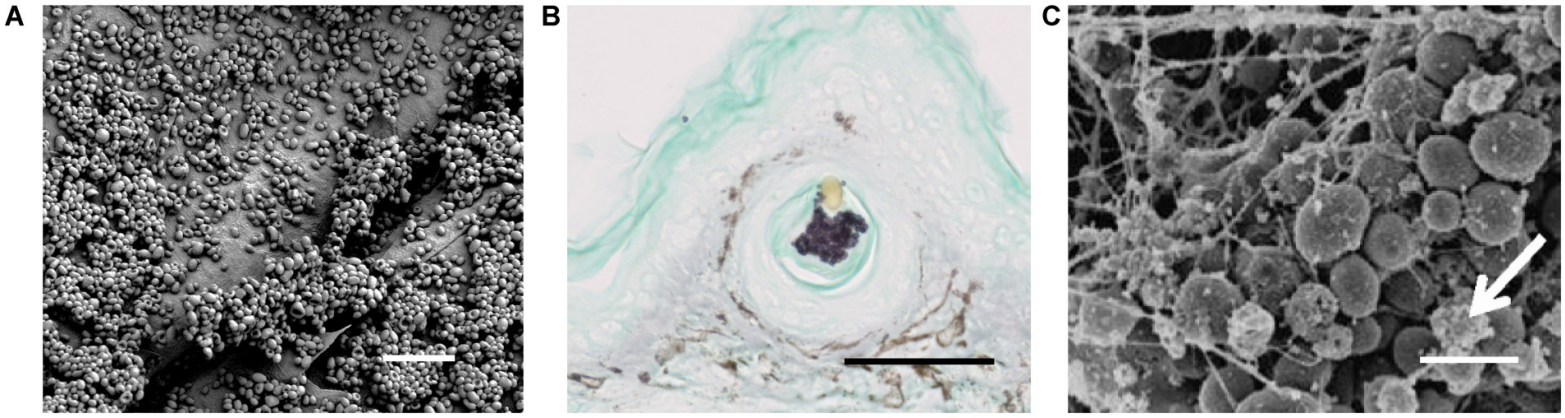
Skin colonization and catheter-associated infection models for *C*. *auris*. **(A)** *C*. *auris* growing on the surface of porcine skin ex vivo, reproduced from Horton and colleagues [37]; measurement bar represents 10 μm. (**B**) *C*. *auris* replicating in the hair follicle of an immunosuppressive murine model of *C*. *auris* skin colonization, reproduced from Huang and colleagues [35]; measurement bar represents 50 μm. (**C**) *C*. *auris* growing as a biofilm on the luminal surface of a rat vascular catheter, reproduced from Dominguez and colleagues; measurement bar represents 5 μm [62].

Animal models of catheter-associated infection mirror clinical observations of high-burden biofilm growth for *C*. *auris* in this setting (Fig 2 and Table 1). Using an indwelling rat jugular venous catheter model, Dominguez and colleagues found multiple *C*. *auris* clinical isolates to propagate as biofilms on luminal catheter surfaces [62]. Some isolates appeared to form even thicker biofilms with more extracellular matrix in vivo than in vitro. Vila and colleagues examined 2 *C*. *auris* clinical isolates in a murine model of subcutaneous catheter fragment implants [27]. The group included one isolate with high biofilm capacity under in vitro conditions and one with low capacity biofilm formation. Surprisingly, both isolates replicated to burdens beyond *C*. *albicans* on catheters in vivo. This suggests that some in vitro conditions may not be representative of biofilm formation in the context of infection and colonization. Although *C*. *auris* isolates exhibit heterogeneity in the capacity for biofilm formation, biofilms formed in standard laboratory media on plastic have generally been less dense in comparison to *C*. *albicans* [27,32,41,63]. However, biofilm formation within models mimicking the skin microenvironment and catheterization have yielded increased recovery for *C*. *auris* compared to *C*. *albicans* [27,41,47]. Catheter-associated biofilm formation by *C*. *auris* further complicates treatment due to increased tolerance of multiple antifungals that occurs during biofilm formation [32,62,64].

**Table 1:** In vivo and ex vivo skin and catheter colonization models.

Infection model	In vivo *C*. *auris* findings	Strain(s)	Clade(s), country of isolation	Comparisons and notes	Ref
Rat central venous catheter	Biofilm formation, drug sequestration	B11104, B11203 (AR-0387), B11211, B11219, B11220 (AR-0381), B11221 (AR-0383), B11785, B11799, B11801, B11804	Pakistan, Colombia, India, Japan, South Africa, Colombia	Biofilm formation on in vivo catheters, deposition of extracellular matrix	[62]
Immunocompetent BALB/c mice, skin topical application	Higher fungal burdens in lung and brain tissue for filamentous cells	BJCA001	Clade I, China	Morphologies include typical yeast (not induced to form filaments at low growing temperatures), filamentation-competent yeast, and filamentous cells	[69]
Ex vivo porcine skin	Biofilm formation and desiccation resistance in skin niche conditions	B11804, B11220 (AR-0381), B11221 (AR-0383), B11801, B11203 (AR-0389), B11219, B11211, B11104, B11799, B11785	Clades I, II, III, IV, Columbia, Japan, South Africa, India, and Pakistan	*C*. *auris* had greater viability after desiccation than *C*. *albicans*; *C*. *auris* biofilms were denser on porcine skin model grown in sweat media than *C*. *albicans* grown with same conditions	[37]
BALB/c mice, subcutaneous catheter	Adhesion to catheter, high replication	AR-0382 and AR-0387	Clade I	Increased recovery of low-biofilm and high-biofilm *C*. *auris* strains compared to *C*. *albicans*	[27]
In vivo guinea pig		MRL 35368		Oral dosing of ibrexafungerp reduced the skin *C*. *auris* fungal burden compared with untreated controls	[39]
Wild-type and immunodeficient C57BL/6 and C57BL/10 mice, topical application on dorsal skin and pinna areas	Ability to reside in deep compartments of the skin for extended periods of time	AR-0387, AR-0381, AR-0383, AR-0385, NIH clinical center	Clades I, II, III, IV	IL-17R pathway limits *C*. *auris* skin colonization	[35]
Ex vivo porcine skin	Resistance to killing by chlorhexidine on the skin	B11203 (AR-0389)	Clade I, India	Addition of isopropanol, tea tree oil, or lemongrass oil could augment the activity of chlorhexidine	[36]
Ex vivo human and porcine skin	High biofilm burden on skin surface	B11203 (AR-0389), B11219, B11211, B11104, B11804, B11801, B11785, B11799, B11220 (AR-0381), B11221 (AR-0383)	Clades I, II, III, IV, Columbia, Japan, South Africa, India, and Pakistan	*C*. *auris* colonizes the skin to greater biofilm burdens than other *Candida* species, including *C*. *haemulonii*	[47]
Rat central venous catheter and ex vivo human skin	Increased biofilm colonization on skin and catheter surface with expression of adhesins	AR-0382, AR-0387, AR-0382 Δ*scf1*/Δ*iff4109*, AR-0382 *pTEF1-SCF1*	Clade I	Overexpression of *SCF1* in the AR-0387 was sufficient to induce skin and catheter colonization of this isolate	[59]

## Secreted aspartyl proteases (SAPs) and cellular morphology

Like other *Candida* spp., *C*. *auris* produces secreted aspartyl proteases (SAPs), which contribute to virulence through the cleavage of host proteins. SAPs can alter adhesive properties, promote tissue invasion, influence immune responses, and disrupt complement signaling [51,52,65,66]. Maybe not surprisingly, SAP production varies among *C*. *auris* isolates and environmental conditions [43,67]. For example, Fan and colleagues examined SAP activity for 2 isolates from China (Clade I and Clade III) [67]. The Clade III strain produced the most SAP activity at 37°C with lesser at lower temperatures (30°C and 25°C), while the Clade I strain produced similar amounts across the temperature range [67]. The Clade I strain was more virulent in *Galleria* and murine models of infection. However, a specific link to virulence is not entirely clear, as many differences were noted between the strains. In addition to the different clade designation, the Clade III isolate exhibited an aggregation phenotype and increased drug resistance, while the Clade I strain did not aggregate. The mechanisms of how SAP production influences *C*. *auris*–host interactions remains unclear. Considering the lower temperature of the skin surface, the authors note that decreased SAP production at these temperatures may be beneficial to dampen immune responses during long-term persistence on skin. In addition, *C*. *auris* has been noted to produce SAPs at high temperatures as high as 42°C [43]. This is consistent with its observed thermotolerance and suggests a role for SAPs in warm environmental conditions [68].

In addition to genetic factors, SAP production appears to be further influenced by cellular morphology and biofilm formation [46,69]. *C*. *auris* typically grows in yeast form, with more rare reports of filamentous structures [43]. However, Yue and colleagues described how passage of *C*. *auris* through a mouse via a tail vein bloodstream infection model yielded distinct morphologies [69]. These included typical yeast forms, filament competent forms, and filamentous morphologies. Interestingly, the yeast-filamentous transition was shown to be heritable, while the filament competent-filamentous transition was nonheritable and dependent on temperature. The finding that lower temperatures (20°C and 25°C) promoted filamentous growth for *C*. *auris* is distinct from *C*. *albicans*, where hyphal growth is triggered by higher temperatures. They examined the impact of morphology on virulence using a systemic infection model of BALB/c mice and found similar viable burdens in the kidney, spleen, and liver, but greater burdens from the brain and lungs of a mice infected with the filamentous form [69]. Upon examination of SAP production at 37°C, both the filament competent and filamentous phenotypes displayed more activity than the yeast forms, suggesting that SAPs may be contributing to the virulence. This was temperature dependent, as at low temperatures the yeast exhibited more activity than the other forms. Other work has shown the presence of elongated, aggregated, and mixed morphologies among *C*. *auris* clinical isolates independent of passage through a mammalian host [70]. Similar to the murine studies, the isolates with filamentous morphology appear more virulent in a *Galleria mellonella* infection model.

## Extracellular vesicle formation by *C*. *auris*

Diverse fungal species, including *C*. *albicans*, secrete extracellular vesicles (EVs), structures of lipid bilayer-enclosed cargo that modulate morphologic changes, host interactions, and drug resistance [71,72]. Like *C*. *albicans*, *C*. *auris* also produces vesicles during planktonic and biofilm modes of growth; however, some of the cargo and properties differ [72,73]. Zamith-Miranda and colleagues examined *C*. *auris* vesicles produced during planktonic growth, comparing them to *C*. *albicans* vesicles. While vesicles from both species contained sterols, RNA, protein, and lipids, the specific contents analyzed by proteomics and lipidomics varied significantly, suggesting that their activities may diverge as well. In functional analysis, the group found that *C*. *auris* EVs could augment fungal adhesion to epithelial cells, while *C*. *albicans* EVs did not. For one (of two) of the *C*. *auris* strains tested, EV treatment enhanced replication and survival after phagocytosis by a murine-derived macrophage line. EVs from the 2 *C*. *auris* isolates were found to stimulate murine bone marrow–derived dendritic cells (BMDCs) by increasing expression of MHCII and costimulatory molecules in a pattern similar to *C*. *albicans* [73]. Comparison of an azole-resistant isolate to an azole-susceptible strain revealed differences in both content and functional activity, suggesting that EVs may be altered in the setting of drug resistance or that they may vary broadly across strains [73]. In other work, *C*. *auris* EVs were shown to augment *C*. *auris* survival in the presence of amphotericin B, while *C*. *albicans* EVs did not, further highlighting differences in EV activities between the species [74].

Zarnowski and colleagues analyzed *C*. *auris* EVs produced during biofilm growth, comparing their enclosed cargo to other *Candida* spp. [72]. The monosaccharide analysis revealed the presence of mannan and glucan in relatively similar ratios across EVs collected from *C*. *albicans*, *C*. *parapsilosis*, *C*. *tropicalis*, *C*. *glabrata*, and *C*. *auris*. The mannan:glucan ratio was also consistent with the ratio of these polysaccharides in the extracellular matrix of *C*. *albicans* biofilms, as EVs have been shown to deliver and deposit a mannan–glucan complex capable of antifungal sequestration [75]. On proteomic analysis, they found high variability across the EV proteomes but identified a set of cargo proteins common to all EVs. Genetic disruption of these proteins impacted biofilm-associated drug tolerance with the exogenous addition of EVs reversing the phenotype across species. This suggests that *C*. *auris* EVs may act cooperatively with other *Candida* spp. In subsequent work examining adhesion, competitive interactions for EVs across species were identified [76]. Little is known about the interaction of *C*. *auris* biofilm EVs with the host, but considering studies with planktonic EVs, they may influence adhesion to host and/or immune recognition [73].

## Modeling of systemic *C*. *auris* infection

Many studies have analyzed *C*. *auris* virulence utilizing a variety of animal models, including mice, zebrafish, *G*. *mellonella*, and *Caenorhabditis elegans* (Tables 2 and 3). Murine models have primarily included pharmacologically immunosuppressed animals, but complement 5-deficient mice have been used as well [77]. *C*. *auris* exhibits increased mortality and higher fungal burden when compared to related and non-*albicans* species such as *C*. *haemulonii*, *C*. *glabrata*, and *C*. *parapsilosis*, in murine, zebrafish, and *Galleria* models [32,54,68,78–81]. However, there is some variability in mortality overserved for individual *C*. *auris* isolates in both murine and *Galleria* models [32,54,67,82]. Studies have been more mixed for comparison of *C*. *auris* and *C*. *albicans*. Murine models have generally observed lower mortality for *C*. *auris*, while *Galleria* and zebrafish studies have reported comparable or increased virulence compared to *C*. *albicans* [26,32,44,54,68,78–81,83,84]. In a *Galleria* model, higher mortality has been observed for isolates that do not aggregate compared to aggregating strains [32,54]. Studies using this model have also found variability in virulence based on the body site of isolate collection, with higher mortality for bloodstream isolates compared to urine or respiratory samples [44]. As patients with *C*. *auris* bloodstream infections are anticipated to have the same strains as colonizers of the skin, respiratory tract, and/or urine, this finding suggests a possible phenotypic alteration or switch during human bloodstream infection, as has been described in mice [44,69].

**Table 2:** Vertebrate models of invasive disease.

Infection model	In vivo *C*. *auris* findings	Strain(s)	Clade(s), country of isolation	Comparisons and notes	Ref
Immunosuppressed BALB/c mice, disseminated candidiasis (cyclophosphamide-treated)	Burden in kidney, forming aggregates	TA005-14	n/a, Israel	Increased fungal burden and mortality compared to *C*. *hauemulonii*, less than *C*. *albicans*	[68]
Immunocompetent BALB/c mice, disseminated candidiasis	Higher fungal burden in spleen compared to *C*. *albicans*	BJCA001	Clade I, China	No lethality in mouse	[43]
Immunocompetent ICR mice, disseminated candidiasis	High burdens in kidney, also recovery from spleen, liver, and lungs	VPCI 479/P13 and VPCI 510/P14	n/a, India	Comparable mortality and fungal burden to *C*. *albicans*	[78]
Zebrafish, larval hindbrain injection	Low neutrophil recruitment, impaired NETosis	B11203 (AR-0389)	Clade I, India	*C*. *albicans* triggers neutrophil responses in zebrafish to a greater extent than *C*. *auris*	[85]
Immunocompetent BALB/c mice, intraperitoneal injection, intravenous injection	Higher fungal burdens in lung and brain tissue for filamentous cells	BJCA001	Clade I, China	Morphologies include typical yeast (not induced to form filaments at low growing temperatures), filamentation-competent yeast, and filamentous cells	[69]
ICR CD-1 immunosuppressed (cyclophosphamide and cortisone acetate treated), disseminated candidiasis and vaccination	Fungal burdens in kidney and heart; biofilm formation; resistance to killing	CAU-09 (AR-0389)	Clade I	NDV-3A vaccine reduced mortality in *C*. *auris*-infected mice	[93]
C5-deficient A/J mice, disseminated candidiasis (cyclophosphamide-treated)	Rapid proliferation in target organs and rapid fatal response	AR-0381 and AR-0386	Clade II, Clade IV	NE^−/−^ and C57BL/6J mice survived *C*. *auris* infection, even upon cyclophosphamide treatment	[95]
Immunosuppressed BALB/c mice	n/a	Ca432 and Ca446	n/a, Colombia	Similar lethality to *C*. *haemulonii* species complex	[80]
Immunocompromised neutropenic BALB/c mice, disseminated candidiasis (cyclophosphamide-treated)	High burdens in heart and kidneys	*C*. *auris* 196, *C*. *auris* 164, NCPF 8984, CBS 12373, NCPF 13042, *C*. *auris* 204, *C*. *auris* I-24, *C*. *auris* 16565,	South Asian (Oman, England), East Asian (Japan, Korea), South African (England), South American (Israel, Chicago)	High lethality strains were similar to *C*. *albicans* in heart and kidney burden; intermediate and low lethality strains had lower burdens	[82]
Immunocompetent A/J mice and immunosuppressed (cyclophosphamide treated) C57BL/6 mice, disseminated candidiasis	Disseminated spread to tissues including kidney, brain, and heart	AR-0386 and AR-0389	Clade I and IV	Monoclonal antibodies against β-1,2-mannotriose, hyphal wall protein 1, and phosphoglycerate kinase 1 enhanced survival and decreased target organ fungal burden	[77]
Zebrafish, larval hindbrain injection	Low neutrophil recruitment, high burden	B11203 (AR-0389)	Clade I, India	Disruption of *PMR1* and *VAN1* causes increased neutrophil recruitment and lower fungal burden compared to WT	[96]
Zebrafish, larval swim-bladder inoculation	Induce proinflammatory cytokine genes and down-regulate recruitment genes	*C*. *auris* SI-18-CAU-HEM	n/a, Thailand	Highest lethality and burden for *C*. *auris* compared to *C*. *haemulonii* and *C*. *albicans*	[79]
Immunocompetent BALB/c mice, intravenous injection	Greater production of SAPs at 25 and 30°C, greater copies of the active Zorro3 retrotransposon	BJCA001 and BJCA 002	Clade I and III, China		[67]
C57BL/6 immunocompetent mice, disseminated candidiasis	Low innate immune cell recruitment to kidneys and spleen, blunted proinflammatory cytokine response	BJCA001	Clade I, China	Decreased immune cell recruitment compared to *C*. *albicans*	[87]
ICR CD-1 immunosuppressed (cyclophosphamide and cortisone acetate treated), disseminated candidiasis	Fungal burdens in kidney and heart	CAU-09 (AR-0389)	Clade I	HX01 mAb reduced mortality in mice infected with *C*. *auris*	[92]
ICR CD-1 immunosuppressed (cyclophosphamide and cortisone acetate treated), disseminated candidiasis	Increased mortality and higher fungal burdens in the kidneys and heart with expression of adhesins	AR-0382, AR-0387, AR-0382 Δ*scf1*/Δ*iff4109*, AR-0382 *pTEF1-SCF1*	Clade I	Loss of adhesins SCF1 and IFF4109 increased survival and reduced dissemination to the kidneys and heart	[59]

**Table 3:** Invertebrate models of invasive disease.

Infection model	In vivo *C*. *auris* findings	Strain(s)	Clade(s), country of isolation	Comparisons and notes	Ref
*Galleria mellonella*	Psuedohyphae production, dissemination in vivo	Clinical isolates from National Health Service hospitals in the United Kingdom	n/a, United Kingdom	Aggregating isolates showed less lethality than non-aggregating	[32,54]
*Galleria mellonella*	Lysis of hemocytes, high fungal burden, tissue invasion	Ca432, Ca446, Ca386, Ca881, Ca885	n/a, Colombia	Higher lethality than *C*. *haemulonii* species complex	[80]
*Galleria mellonella*	Melanization, decreased activity scores, cocoon formation	*C*. *auris* DSM 21092 Cau40, Cau63	n/a, Colombia	Clinical strains were more virulent than reference strains, no difference in mortality between Agg vs. non-Agg strains	[84]
*Galleria mellonella*	Increased growth rate or secretion of virulence factors at 37°C compared with 30°C	BJCA001, RICU13	Clade I, III	Typical yeast and aggregating forms less virulent than filamentous and nonaggregating forms	[70]
*Galleria mellonella*	Melanization, filament and pseudohyphal formation, and high tissue invasiveness	2018-1-124819, Cj104, Cj98, 253107, 182482, 312755, Cj197, Cj198, Cj175, Cj173	n/a, Spain	*C*. *auris* isolates less virulent than *C*. *albicans* but more virulent than *C*. *parapsilosis*; aggregative phenotypes less pathogenic than nonaggregative	[81]
*Galleria mellonella* and *C*. *elegans*		Clinical blood, oropharyngeal, and urine isolates from the Hospital Universitario y Politécnico La Fe of Valencia, Spain and the Institut für Hygiene und Mikrobiologie, Würzburg, Germany	n/a, Spain and Germany	*Galleria* model showed variability in virulence based on site of *C*. *auris* isolation with highest virulence in blood isolates	[44]
*Galleria mellonella*	Greater production of SAPs at 25 and 30°C, greater copies of the active Zorro3 retrotransposon	BJCA001 and BJCA 002	Clade I and III, China		[67]
*Galleria mellonella*	Invades respiratory system, high immunogenic activity causing inflammatory nodules of aggregated plasmatocytes	CJ175, CJ101	Clade III, n/a	Less pseudohyphae production than *C*. *albicans* and *C*. *glabrata*; unique tropism for *C*. *auris*	[97]

## Immune responses to *C*. *auris* ex vivo, during infection, and on skin

Innate immune responses to *C*. *auris* have been explored using a combination of murine models, zebrafish models, primary leukocytes, and cell lines. Examination of human neutrophil interactions with *C*. *auris* revealed impaired phagocytosis and killing of *C*. *auris* compared to *C*. *albicans* [85]. Poor neutrophil phagocytosis for *C*. *auris* was observed to be conserved across a variety of strains and clades [86]. Unlike the response to *C*. *albicans*, *C*. *auris* induced minimal reactive oxygen species (ROS) and did not trigger the formation of neutrophil extracellular traps (NETs). In other work, Wang and colleagues similarly examined ex vivo *C*. *auris*–neutrophil interactions [87]. Compared to *C*. *albicans*, they observed decreased phagocytosis of *C*. *auris* for both murine and human neutrophils. The impaired phagocytosis also correlated with a decreased ability of neutrophils to kill *C*. *auris*. Using an immunocompetent murine model of disseminated candidiasis, the group also showed diminished neutrophil recruitment to *C*. *auris* in the kidneys and spleen, which correlated with high fungal burdens in these organs when compared to *C*. *albicans*. To assess if the outer cell wall mannan layer may be involved in the poor phagocytosis of *C*. *auris*, Horton and colleagues constructed mutants with disruption of *PMR1*, a putative Golgi-associated ATP-ase pump required for N- and O-mannosylation, and *VAN1*, a putative N-linked mannosyltransferase. Both *C*. *auris* mutants exhibited broad disruption of the cell wall mannan layer. The mutants were more readily phagocytosed and killed by human neutrophils ex vivo and in a larval zebrafish hindbrain injection model [86]. The findings suggest that the outer cell wall layer protects *C*. *auris* from phagocytic responses.

Wang and colleagues further examined the role of mannosylation on innate immune responses, primarily focusing on murine bone marrow–derived macrophages (BMDMs) [87]. In addition to the observed poor neutrophil responses to *C*. *auris* when compared to *C*. *albicans*, they also found a blunted proinflammatory response broadly across innate immune cells in vivo and ex vivo. The *C*. *auris* cell wall contained more mannan than *C*. *albicans*, and the mannan fibrils of *C*. *auris* were twice as long when observed by electron microscopy. To analyze the role of *C*. *auris* mannans, they constructed mutants with disruption of genes with putative involvement in mannosylation, including *PMR1*, (N- and O-mannosylation), *OCH1* (N-mannosylation), and *PMT1* (O-mannosylation). Disruption of either N- or O-mannosylation resulted in proinflammatory responses by BMDMs, consistent with a critical role for mannosylation in *C*. *auris* immune evasion and protection from phagocytosis.

*C*. *auris* appears to utilize multiple mechanisms for immune evasion. A recent study examining macrophage–*C*. *auris* interactions found that *C*. *auris* could escape murine BMDMs after phagocytosis [88]. Following intracellular replication, *C*. *auris* was shown to essentially deplete macrophage glucose concentrations and provoke their killing without induction of inflammasome responses. Corresponding to decreased inflammasome activation, *C*. *auris*-infected BMDMs had decreased production of cytokine IL-1β in comparison to *C*. *albicans*-infected BMDMs.

While some studies have identified immune evasion phenotypes, other work has shown proinflammatory responses for *C*. *auris* [89]. Bruno and colleagues examined *C*. *auris* interactions with human peripheral blood mononuclear cells (PBMCs) and described a greater proinflammatory transcriptional response when compared to PBMCs exposed to *C*. *albicans* [89]. The authors hypothesized that altered mannosylation may play a role and identified a unique M-α-1-phosphate sidechain in the acid-labile portion of *C*. *auris* mannan. They found *C*. *auris* mannans to display lower binding affinity to recombinant human dectin-2 and mannose receptor pattern recognition receptors (PRRs) when compared to *C*. *albicans* mannans. The broad relevance of the mannan linkage is unclear. As other studies have not found this component, it may be dependent on strain or growth conditions [86]. In other work examining *C*. *auris* interactions with PBMCs, Navarro-Arias and colleagues found similar cytokine profiles and phagocytosis rates for PBMCs exposed to either *C*. *auris* or *C*. *albicans* [90]. It appears likely that immune responses vary among *C*. *auris* isolates, phagocytes, and model systems.

Understanding features of the *C*. *auris* cell wall may not only shed light on the pathogenesis of immune responses to *C*. *auris* but also provide insight into treatment strategies. For example, Yan and colleagues noted distinct structures in the mannan of *C*. *auris* that were not found in *C*. *albicans*, including high amounts of β-1,2-linkages in the terminal mannan chains [91]. The alteration was linked to differential IgG binding. In other work, *Candida*-specific antibodies targeting cell wall components (β-1,2-mannotriose, hyphal wall protein, or phosphoglycerate kinase 1) were protective in a disseminated murine model [77]. Monoclonal antibodies targeting the immunogenic cell wall protein of *C*. *auris* (hyphal-regulated protein Hyr1) were shown to prevent biofilm formation, promote opsonophagocytosis, and protect mice from disseminated infection [92]. In addition, the NDV-3A vaccine developed to target the major *C*. *albicans* adhesin Als3 also produces immunity against invasive *C*. *auris* infection in mice [93].

Immune responses to *C*. *auris* on skin have primarily been analyzed using in vivo murine models with skin colonization of the shaved back or ear pinna [35]. Examining sites of colonization in this model, Huang and colleagues showed accumulation of Th17 T cells, specifically in CD4^+^ IL-17A^+^ and CD4^+^ IL-17F^+^ cells [35]. In addition to this CD4^+^ T cell response, an abundance of CD8^+^ T cells producing IL-17A and IL-17F were observed. Upon disruption of the IL-17 receptor signaling pathway, they found increased recovery of *C*. *auris* from the skin, leading to the conclusion that the IL-17 axis participates in limiting *C*. *auris* skin colonization in mice. Previous work has reported the IL-17 response to be critical for controlling *Candida* fungal infections at mucosal surfaces, such as oropharyngeal candidiasis and across the broad spectrum of chronic mucocutaneous candidiasis (reviewed in [94]). It appears similar responses are involved in control of *C*. *auris* on skin.

## Conclusions and future directions

*C*. *auris* is a newly emergent species that persists in the environment and on patient skin despite attempts at decolonization. Hospitalized patients undergoing catheterization and other surgical procedures are at particularly high risk for invasive infection. Cases of *C*. *auris* colonization and infection are on the rise, underscoring the need for effective mechanisms for decontamination and prevention of *C*. *auris* colonization on skin and abiotic surfaces. For invasive infection, development of new therapeutic options and enhancing immune response to *C*. *auris* will be crucial for combatting drug-resistant isolates. Current models for dissecting virulence and host factors involved in *C*. *auris* infection have gained traction for providing insight into *C*. *auris* pathogenesis. However, directly correlating laboratory and animal model findings with human clinical disease will remain a challenge.

A few outstanding questions remain:

- How does *Candida auris* grow effectively in the skin microenvironment?
- How does the human immune system recognize *C*. *auris*, and how can recognition be enhanced?
- How does isolate-specific variation alter *C*. *auris* infection and colonization?
- What are the associations among the biological factors of *C*. *auris* that contribute to virulence? How do these interface with antifungal resistance?
